# Publisher Correction: Socio-spatial equity analysis of relative wealth index and emergency obstetric care accessibility in urban Nigeria

**DOI:** 10.1038/s43856-024-00509-8

**Published:** 2024-05-07

**Authors:** Kerry L. M. Wong, Aduragbemi Banke-Thomas, Tope Olubodun, Peter M. Macharia, Charlotte Stanton, Narayanan Sundararajan, Yash Shah, Gautam Prasad, Mansi Kansal, Swapnil Vispute, Tomer Shekel, Olakunmi Ogunyemi, Uchenna Gwacham-Anisiobi, Jia Wang, Ibukun-Oluwa Omolade Abejirinde, Prestige Tatenda Makanga, Bosede B. Afolabi, Lenka Beňová

**Affiliations:** 1 Faculty of Epidemiology and Population Health, London School of Hygiene & Tropical Medicine, London, UK; 2 School of Human Sciences, University of Greenwich, London, UK; 3 Maternal and Reproductive Health Research Collective, Lagos, Nigeria; 4 Department of Community Medicine and Primary Care, Federal Medical Centre Abeokuta, Abeokuta, Ogun Nigeria; 5 Department of Public Health, Institute of Tropical Medicine, Antwerp, Belgium; 6 Population & Health Impact Surveillance Group, Kenya Medical Research Institute-Wellcome Trust Research Programme, Nairobi, Kenya; 7 Centre for Health Informatics, Computing, and Statistics, Lancaster Medical School, Lancaster University, Lancaster, UK; 8 Google LLC, Mountain View, CA USA; 9 Lagos State Ministry of Health, Ikeja, Lagos Nigeria; 10 Nuffield Department of Population Health, University of Oxford, Oxford, UK; 11 School of Computing & Mathematical Sciences, University of Greenwich, London, UK; 12 Dalla Lana School of Public Health, University of Toronto, Toronto, Canada; 13 Women’s College Hospital Institute for Health System Solutions and Virtual Care, Toronto, Canada; 14 Surveying and Geomatics Department, Midlands State University Faculty of Science and Technology, Gweru, Midlands Zimbabwe; 15 Climate and Health Division, Centre for Sexual Health and HIV/AIDS Research, Harare, Zimbabwe; 16 Department of Obstetrics and Gynaecology, College of Medicine of the University of Lagos, Lagos, Nigeria

**Keywords:** Health services, Public health

Correction to: *Communications Medicine* 10.1038/s43856-024-00458-2, published online 28 February 2024

In the published HTML version of this article, an incorrect version of Figure 4 was provided which included a health facility mapped in an erroneous location. However, the version in the PDF was correct. Figure 4 in the HTML article has now been corrected and matches the figure in the PDF version.

