# Supplementary material for: Socio-spatial equity analysis of relative wealth index and emergency obstetric care accessibility in urban Nigeria
Source: Commun Med (Lond). 2024 Feb 28;4:34. doi: 10.1038/s43856-024-00458-2 (PMC10902387; doi:10.1038/s43856-024-00458-2)
Supplement: Supplementary file 2 — Supplementary Information [file 43856_2024_458_MOESM2_ESM.pdf]

Supplementary Information

Wong et al. 2024. Relative wealth index and accessibility to emergency obstetric care in urban Nigeria: A socio-spatial equity analysis

Table of Contents

Supplementary Table 1. Summary characteristics of the included cities 2
Supplementary Figure 1. S2 cells included in the current analysis for Aba. 3
Supplementary Figure 2. S2 cells included in the current analysis for Abuja. 4
Supplementary Figure 3. S2 cells included in the current analysis for Benin City. 5
Supplementary Figure 4. S2 cells included in the current analysis for Ibadan. 6
Supplementary Figure 5. S2 cells included in the current analysis for Ilorin. 7
Supplementary Figure 6. S2 cells included in the current analysis for Jos. 8
Supplementary Figure 7. S2 cells included in the current analysis for Kaduna. 9
Supplementary Figure 8. S2 cells included in the current analysis for Kano. 10
Supplementary Figure 9. S2 cells included in the current analysis for Lagos. 11
Supplementary Figure 10. S2 cells included in the current analysis for Maiduguri. 12
Supplementary Figure 11. S2 cells included in the current analysis for Onitsha. 13
Supplementary Figure 12. S2 cells included in the current analysis for Owerri. 14
Supplementary Figure 13. S2 cells included in the current analysis for Port Harcourt. 15
Supplementary Figure 14. S2 cells included in the current analysis for Uyo. 16
Supplementary Figure 15. S2 cells included in the current analysis for Warri. 17

Supplementary Table 1. Summary characteristics of the included cities

City	Number of S2 cells	Percentage of S2 cells with no population	Percentage of S2 cells with unavailable travel time^	Public hospitals	Private hospitals	All hospitals	Estimated population 2022	Estimated population 2030	Number of women of childbearing age, 2022	Percentage of women of childbearing age, 2022
Owerri	780	0.0	0.0	2	74	76	945,046	1,282,000	230,314	24.4
Onitsha	2,139	4.3	3.1	1	116	117	1,552,630	2,138,000	397,541	25.6
Aba	2,337	0.0	0.0	2	106	108	1,150,116	1,527,000	377,554	32.8
Abuja	18,989	1.3	0.6	18	53	71	3,652,029	5,119,000	1,095,195	30
Benin City	16,772	30.2	6.5	4	70	74	1,841,084	2,451,000	482,055	26.2
Ibadan	8,844	0.0	0.0	11	152	163	3,756,445	4,956,000	955,580	25.4
Ilorin	2,965	0.3	0.2	7	68	75	1,000,477	1,314,000	283,066	28.3
Jos	8,947	8.2	4.8	6	70	76	942,167	1,236,000	344,494	36.6
Kaduna	21,911	29.8	14.4	5	46	51	1,158,048	1,499,000	513,105	44.3
Kano	8,507	0.1	0.1	16	129	145	4,219,209	5,551,000	1,294,941	30.7
Lagos*	11,393	6.9	3.3	26	770	796	15,387,639	20,600,000	3,402,451	22.1
Maiduguri	2,535	7	2.6	5	21	26	822,337	1,071,000	265,740	32.3
Port Harcourt	11,367	33	10.3	5	79	84	3,324,694	4,595,000	828,146	24.9
Uyo	5,138	1.5	0.7	3	45	48	1,264,636	1,771,000	461,254	36.5
Warri	9,850	14.8	4.2	9	101	110	942,683	1,304,000	567,385	60.2
**Total**	**132,474**	**14.3**	**5.2**	**120**	**1900**	**2020**	**41,959,240**	**56,414,000**	**11,498,821**	**27.4**

+S2 cells with population size 0 were excluded from the analysis. Estimates of city population for 2022 and 2030 were obtained from the UN Urbanization Prospectus, and WoCBA data obtained from WorldPop project. The population of Lagos is disputed, with the State government estimating its 2022 population as 26 million.

^ Accessibility measures were not computed if S2 cell was farther than 50km from any target facility (public, private, or either), or if there was no accessible road path from that S2 cell to a target facility.

Supplementary Figure 1. S2 cells included in the current analysis for Aba.

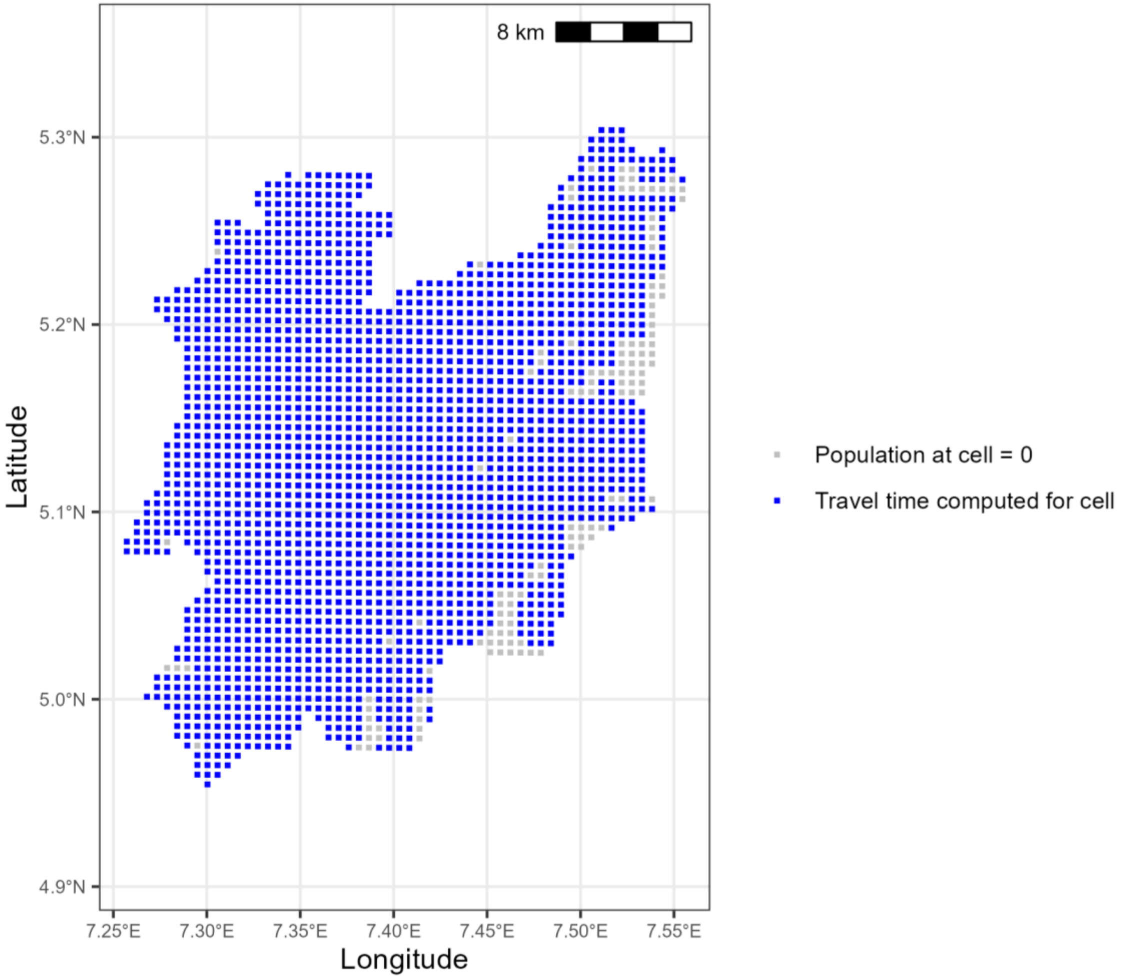

The S2 cells in Aba. Blue cells are include in the current analysis

Supplementary Figure 2. S2 cells included in the current analysis for Abuja.

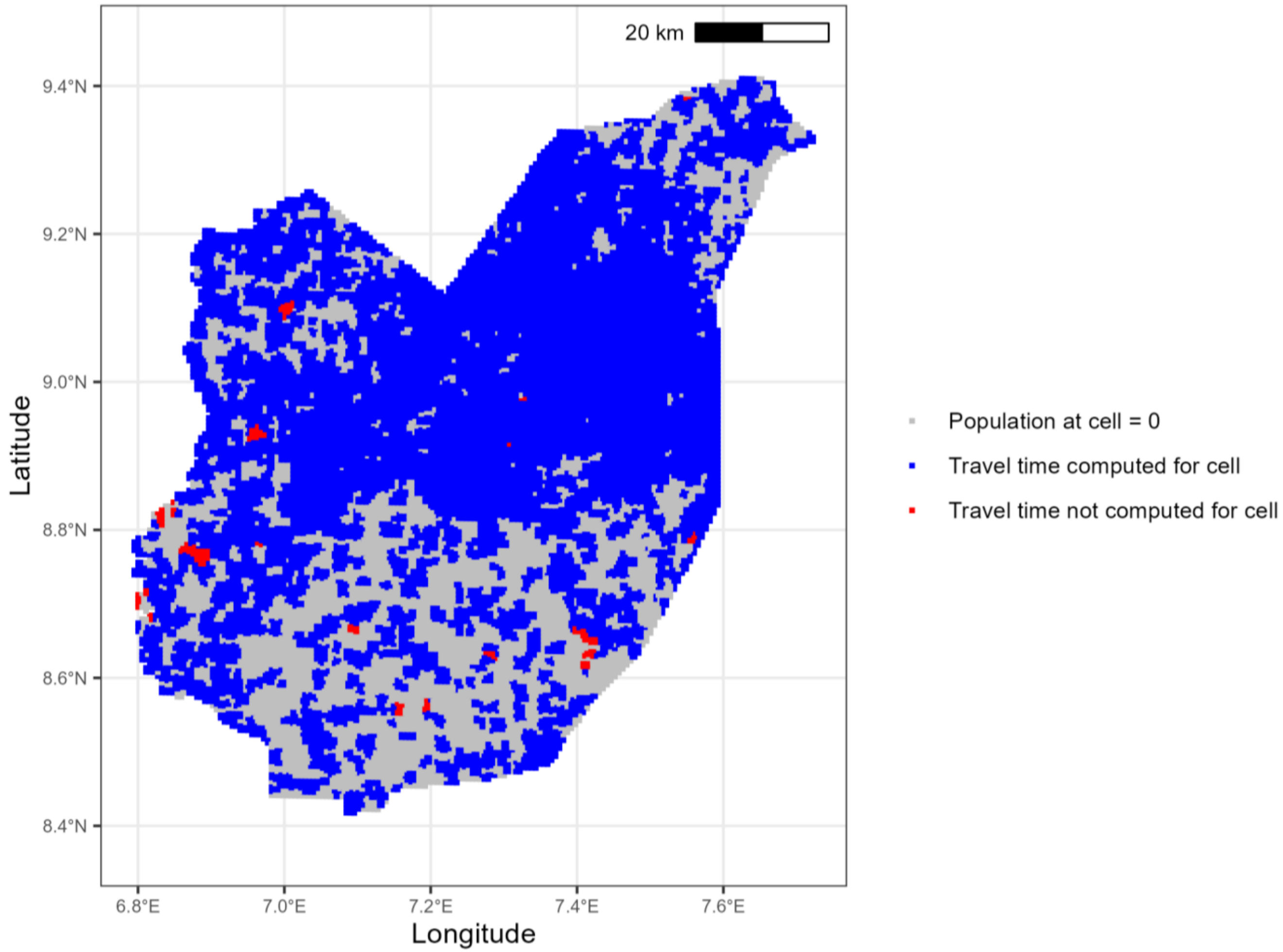

The S2 cells in Abuja. Blue cells are include in the current analysis

Supplementary Figure 3. S2 cells included in the current analysis for Benin City.

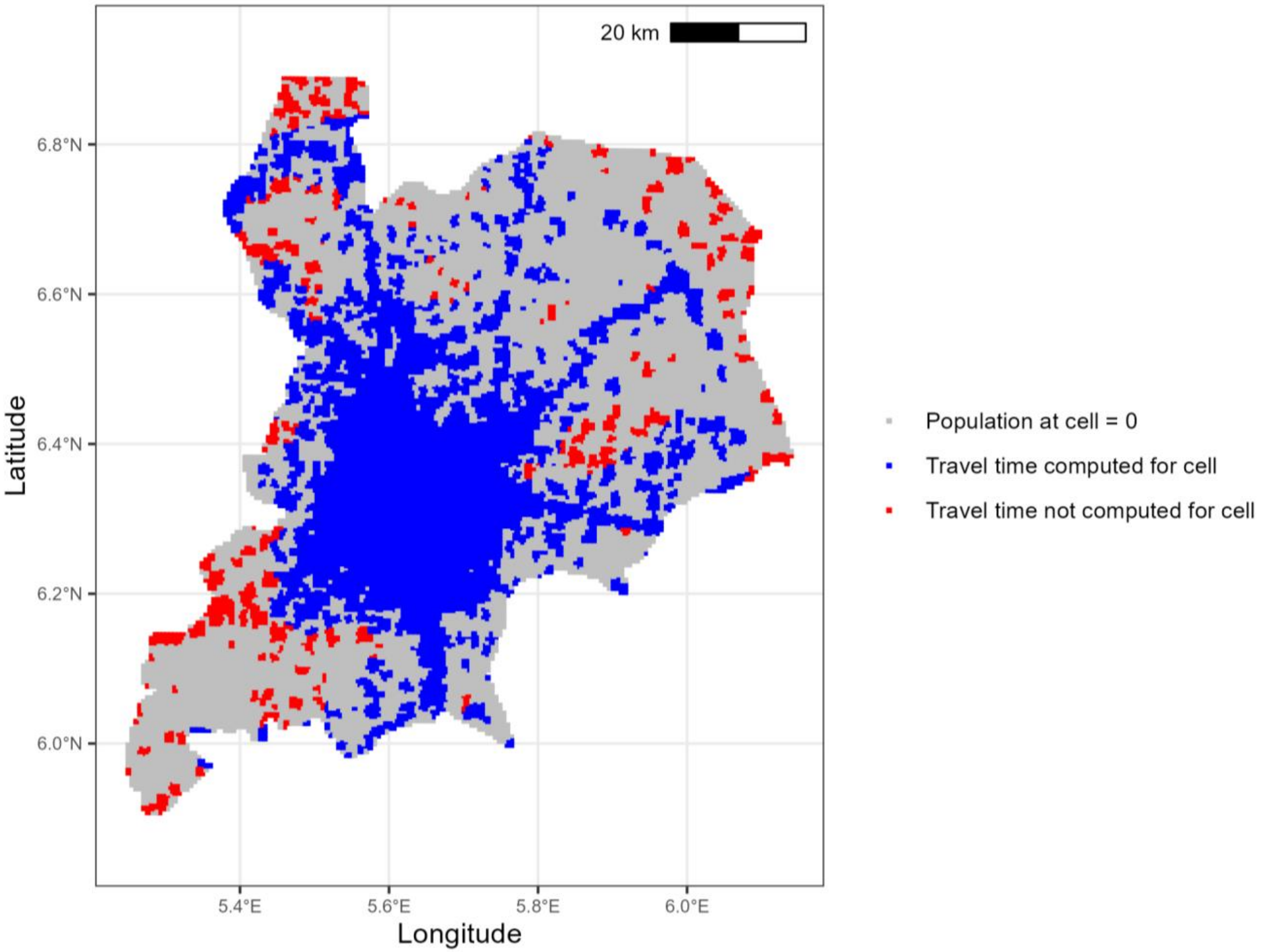

The S2 cells in Benin City. Blue cells are include in the current analysis

Supplementary Figure 4. S2 cells included in the current analysis for Ibadan.

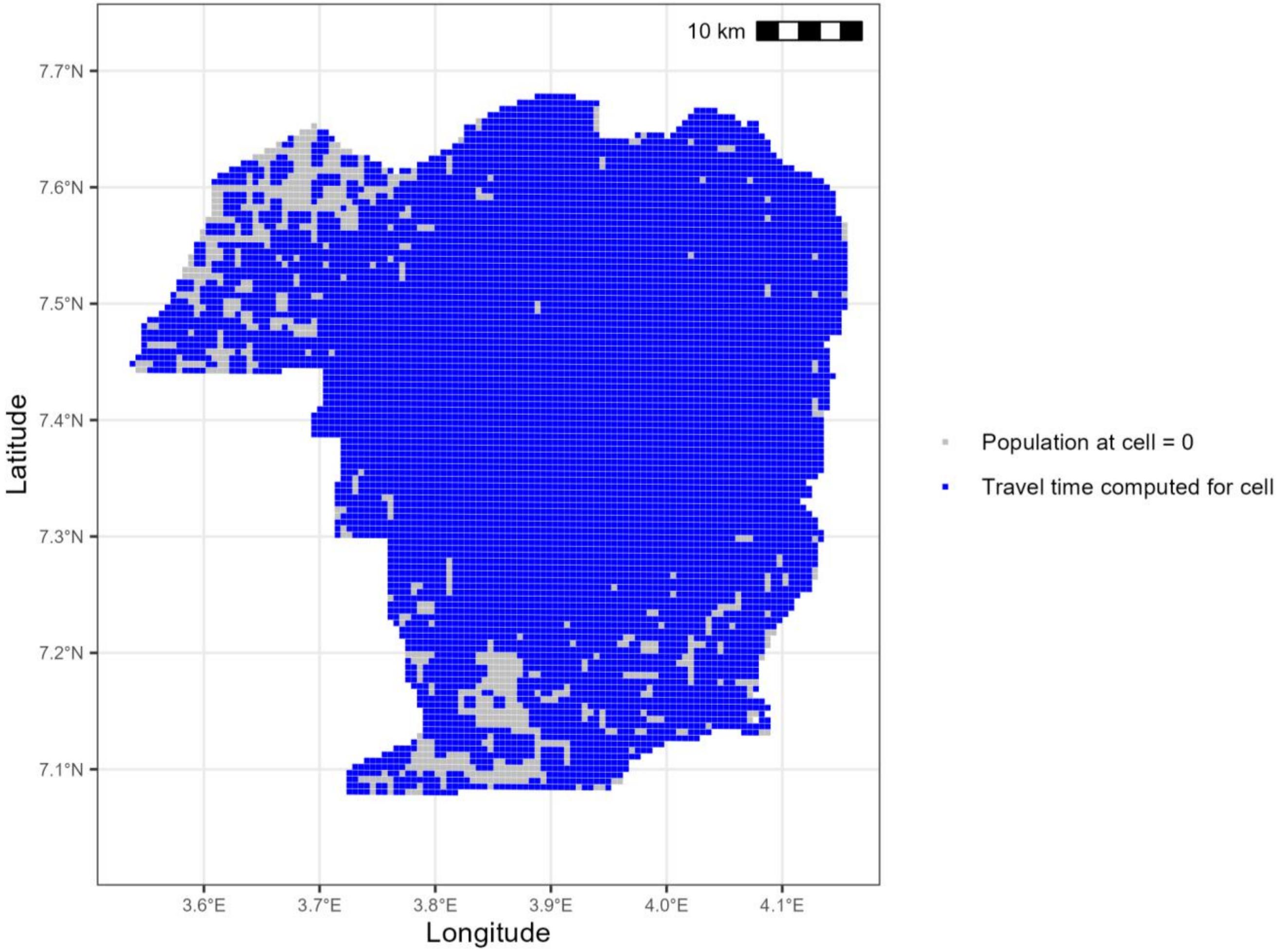

The S2 cells in Ibadan. Blue cells are include in the current analysis

Supplementary Figure 5. S2 cells included in the current analysis for Ilorin.

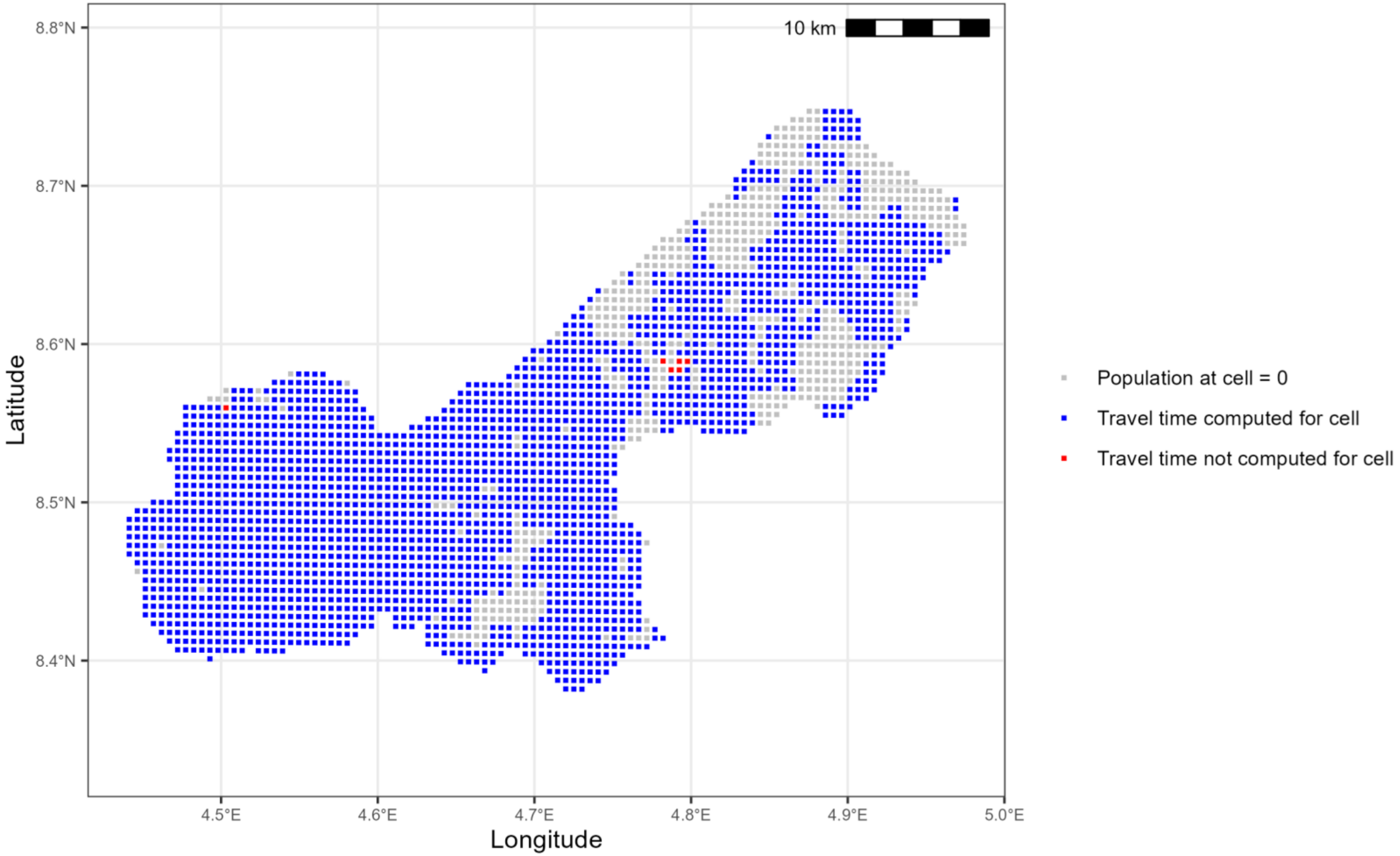

The S2 cells in Ilorin. Blue cells are include in the current analysis

Supplementary Figure 6. S2 cells included in the current analysis for Jos.

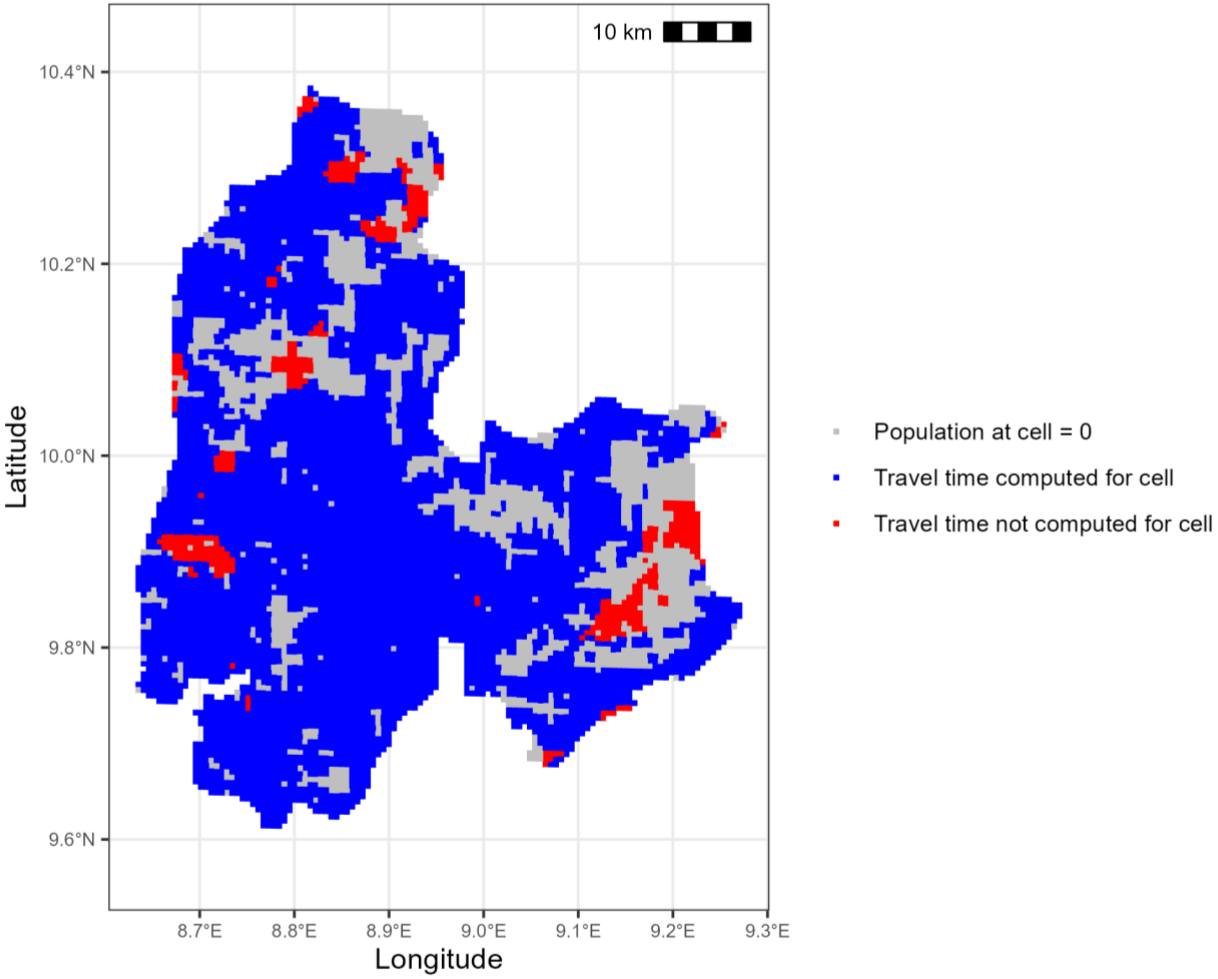

The S2 cells in Jos. Blue cells are include in the current analysis

Supplementary Figure 7. S2 cells included in the current analysis for Kaduna.

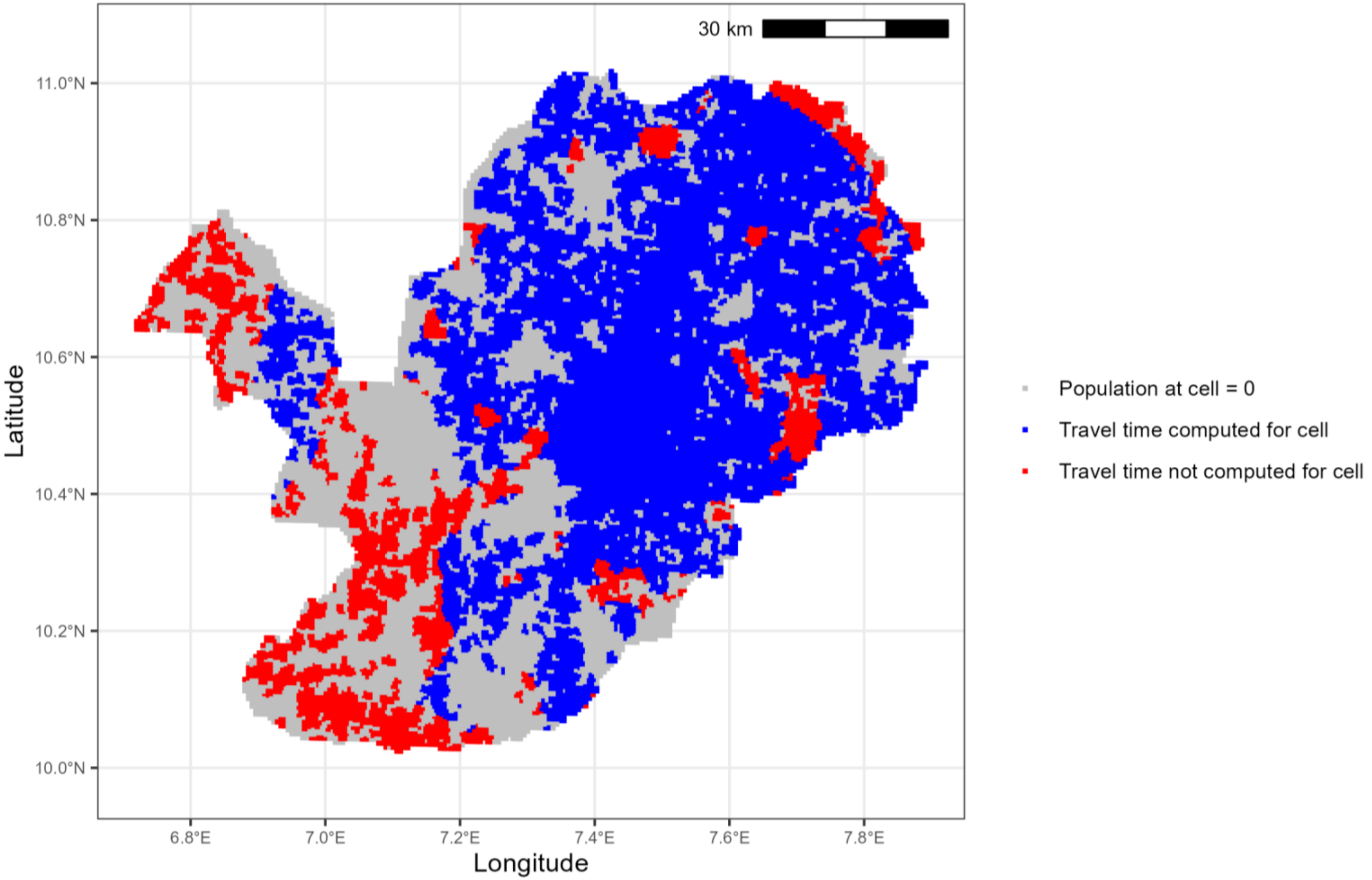

The S2 cells in Kaduna. Blue cells are include in the current analysis

Supplementary Figure 8. S2 cells included in the current analysis for Kano.

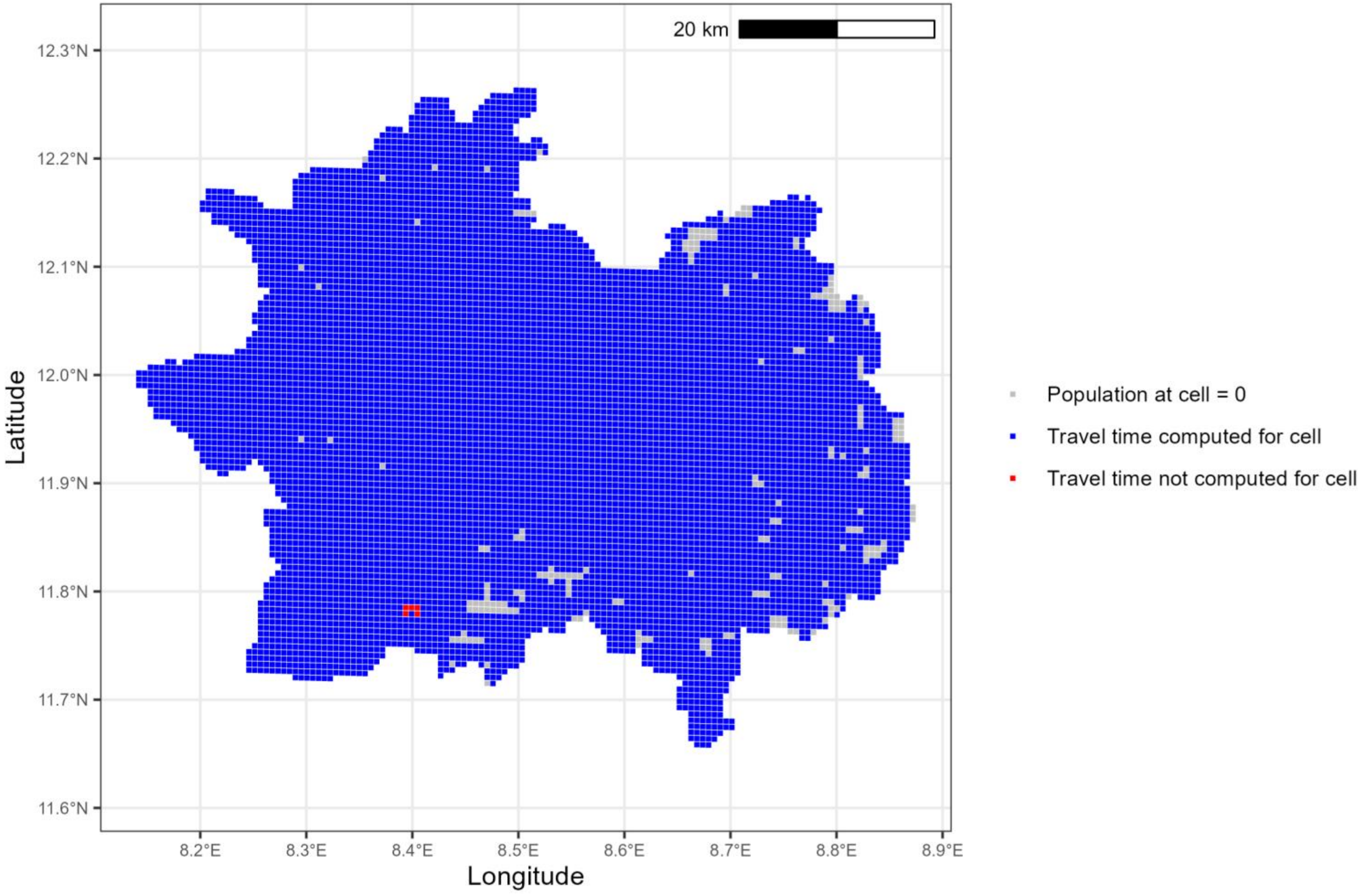

The S2 cells in Kano. Blue cells are include in the current analysis

Supplementary Figure 9. S2 cells included in the current analysis for Lagos.

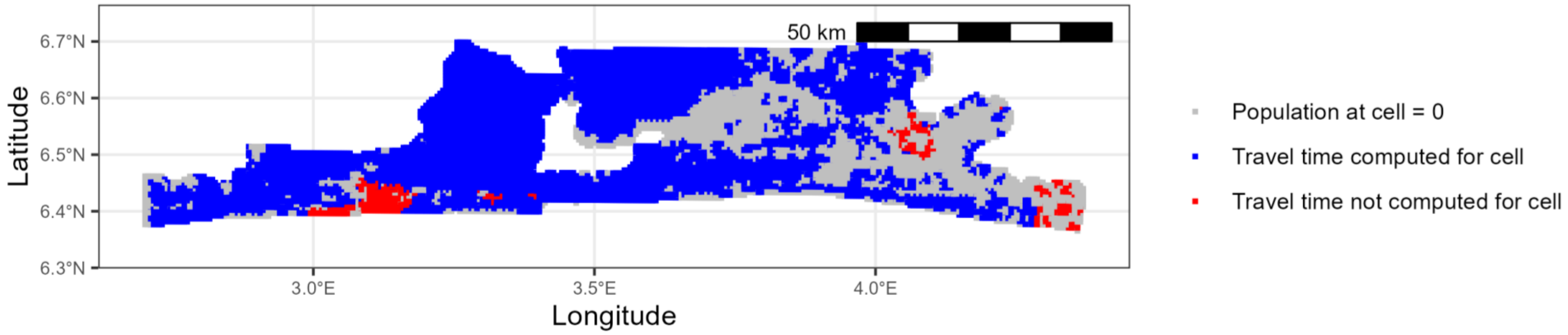

The S2 cells in Lagos. Blue cells are include in the current analysis

Supplementary Figure 10. S2 cells included in the current analysis for Maiduguri.

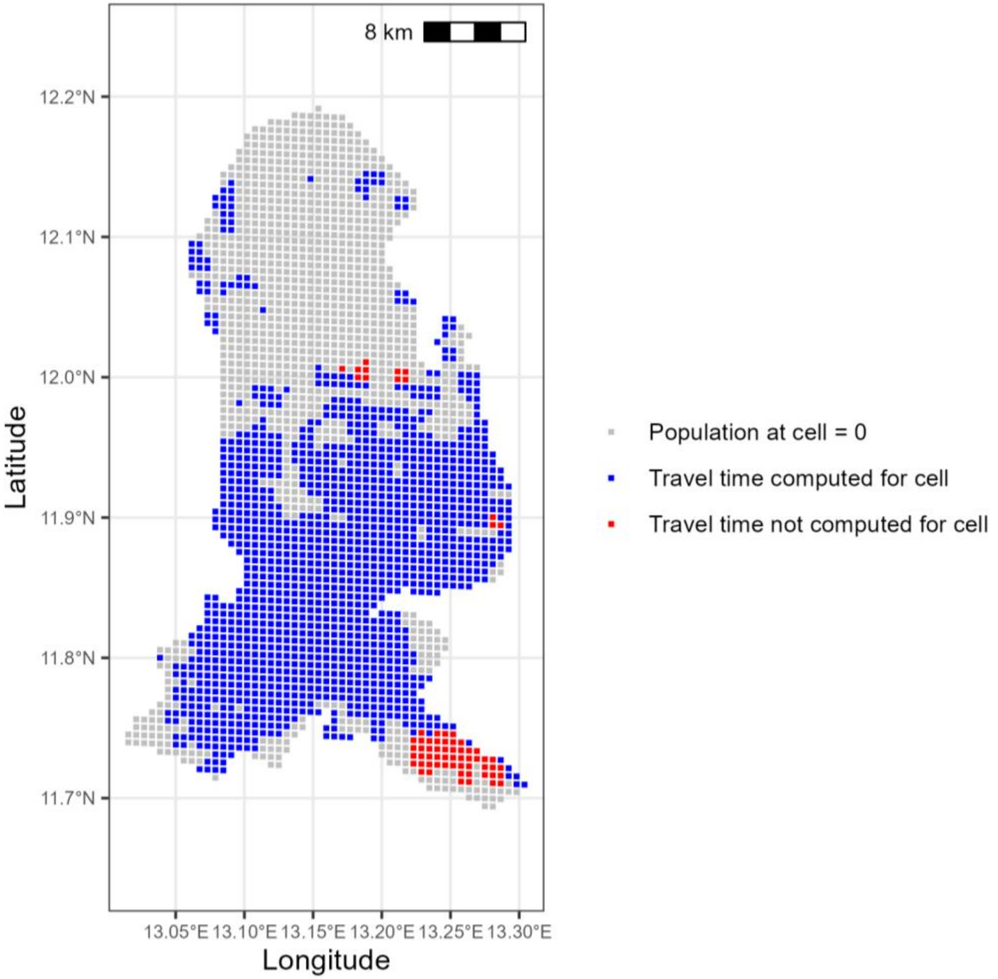

The S2 cells in Maiduguri. Blue cells are include in the current analysis

Supplementary Figure 11. S2 cells included in the current analysis for Onitsha.

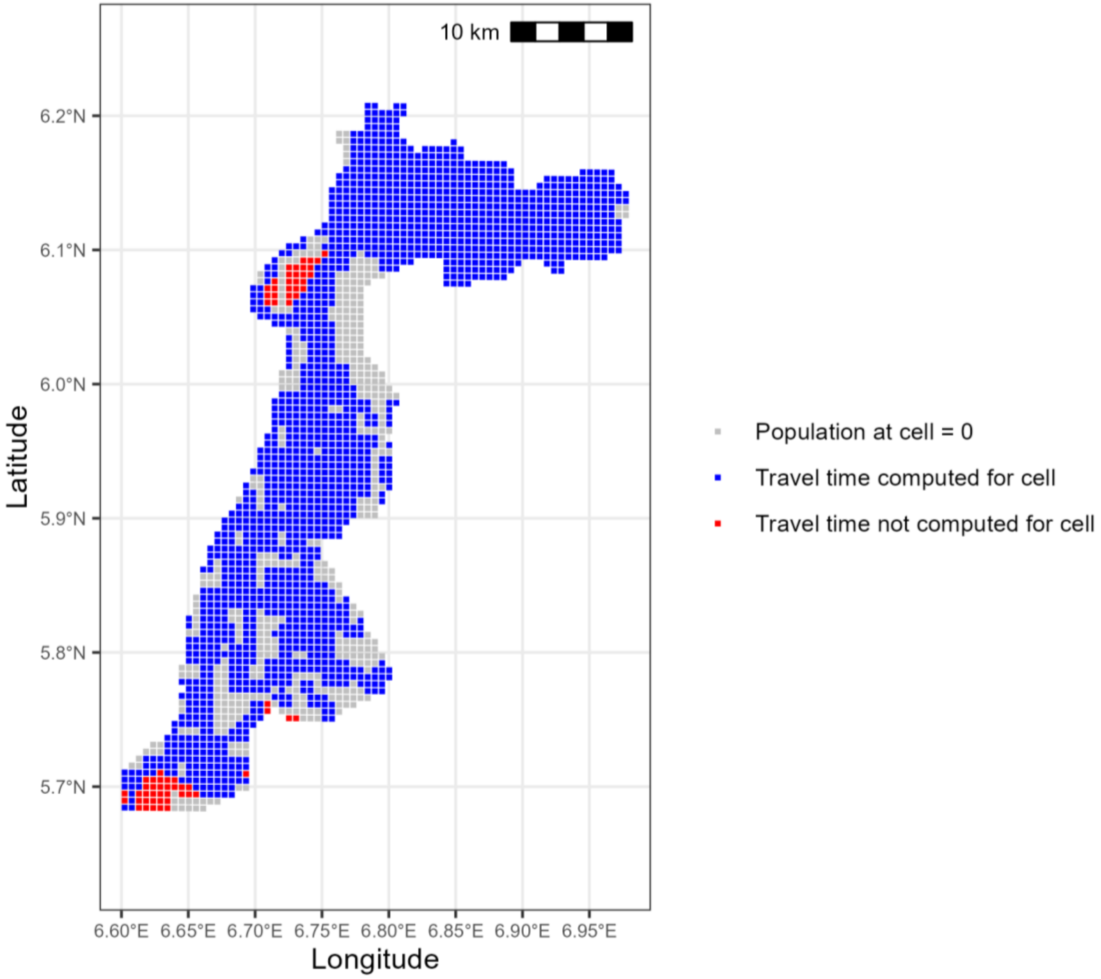

The S2 cells in Onitsha. Blue cells are include in the current analysis

Supplementary Figure 12. S2 cells included in the current analysis for Owerri.

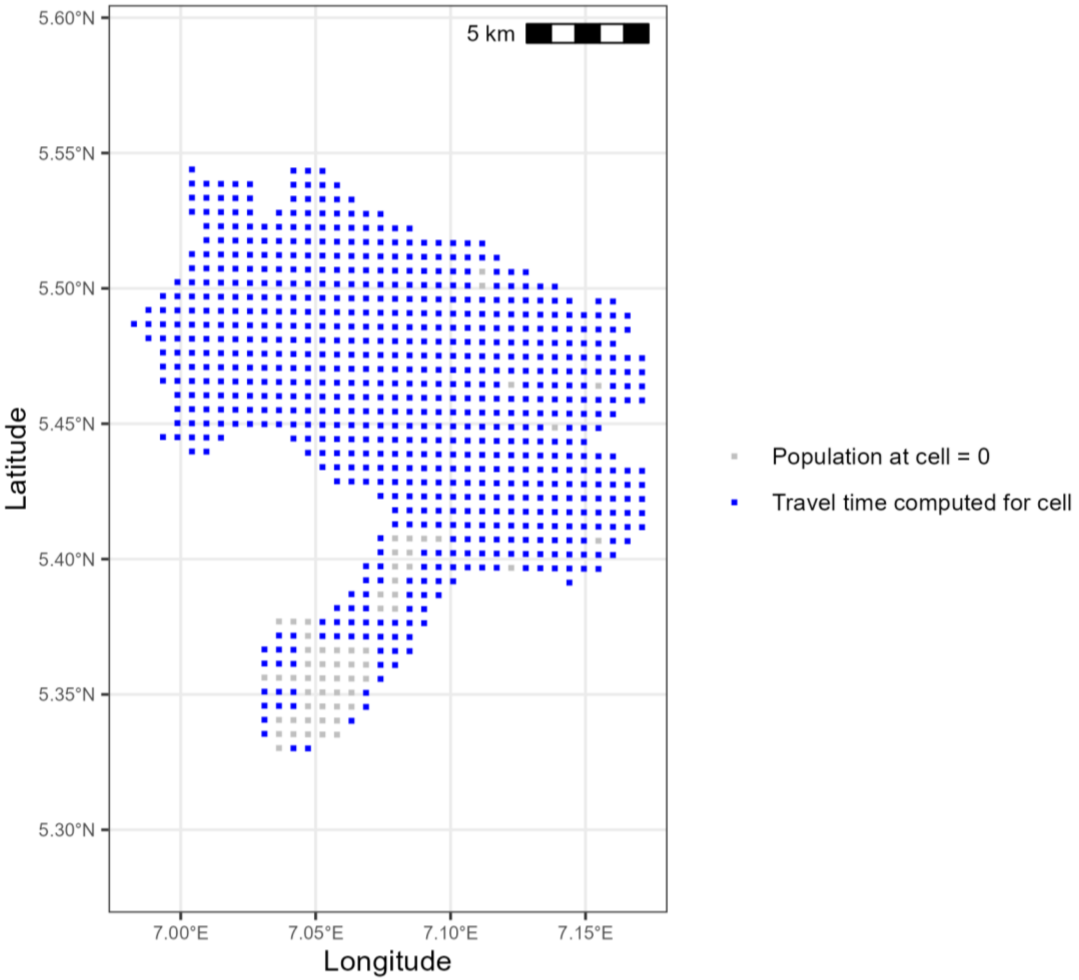

The S2 cells in Owerri. Blue cells are include in the current analysis

Supplementary Figure 13. S2 cells included in the current analysis for Port Harcourt.

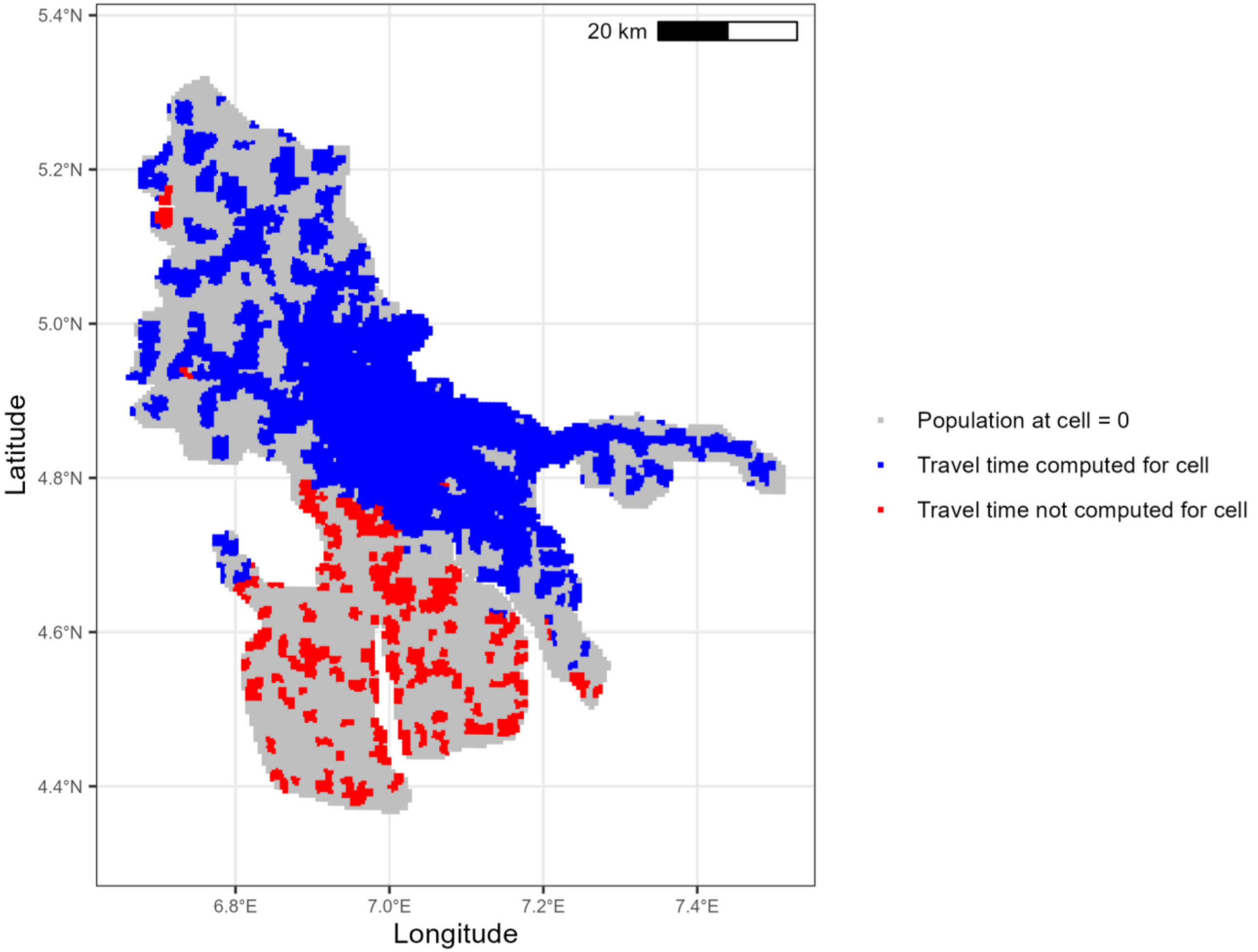

The S2 cells in Port Harcourt. Blue cells are include in the current analysis

Supplementary Figure 14. S2 cells included in the current analysis for Uyo.

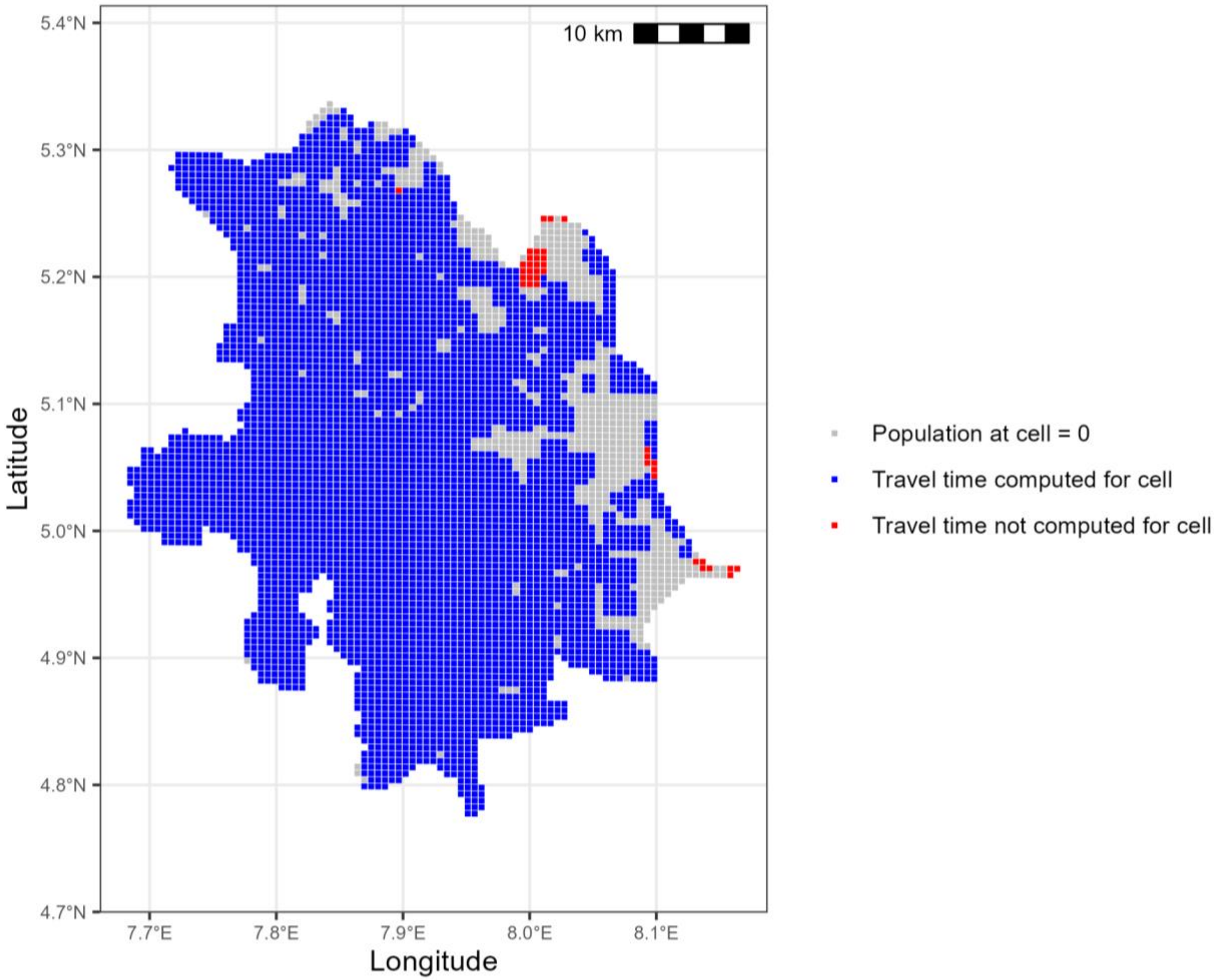

The S2 cells in Uyo. Blue cells are include in the current analysis

Supplementary Figure 15. S2 cells included in the current analysis for Warri.

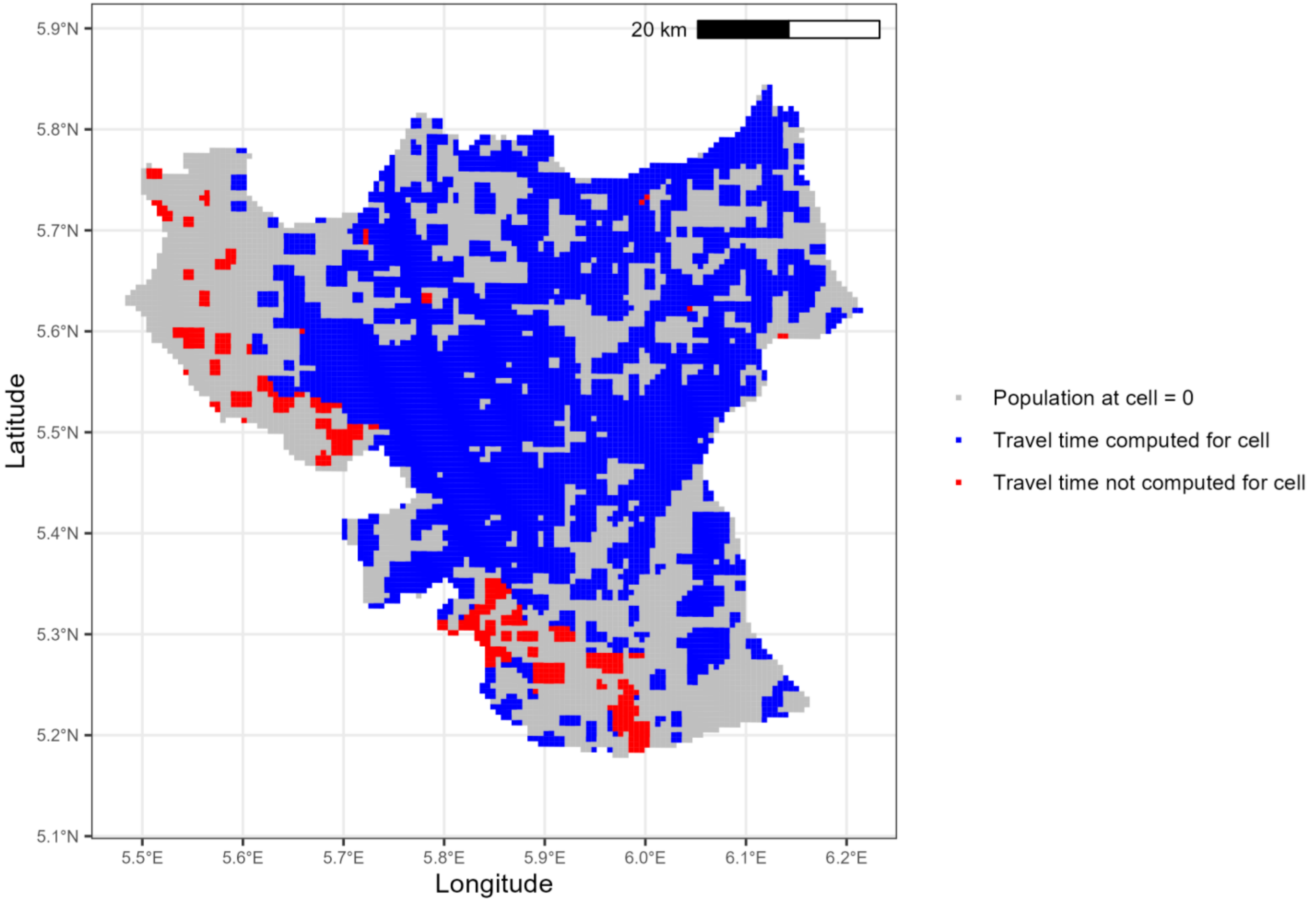

The S2 cells in Warri. Blue cells are include in the current analysis
