# Supplementary material for: Socio-spatial equity analysis of relative wealth index and emergency obstetric care accessibility in urban Nigeria
Source: Commun Med (Lond). 2024 Feb 28;4:34. doi: 10.1038/s43856-024-00458-2 (PMC10902387; doi:10.1038/s43856-024-00458-2)
Supplement: Supplementary file 3 — Description of Additional Supplementary Files [file 43856_2024_458_MOESM3_ESM.pdf]

Description of Additional Supplementary Files

File Name: Supplementary Data 1
Description: Source data for Figures 1-4
