# Supplementary material for: Socio-spatial equity analysis of relative wealth index and emergency obstetric care accessibility in urban Nigeria
Source: Commun Med (Lond). 2024 Feb 28;4:34. doi: 10.1038/s43856-024-00458-2 (PMC10902387; doi:10.1038/s43856-024-00458-2)
Supplement: Supplementary file 5 — Reporting summary [file 43856_2024_458_MOESM5_ESM.pdf]

nature portfolio

Corresponding author(s): Aduragbemi BANKE-THOMAS (COMMSMED-23-0506)

Last updated by author(s): 29/12/23

Reporting Summary

Nature Portfolio wishes to improve the reproducibility of the work that we publish. This form provides structure for consistency and transparency in reporting. For further information on Nature Portfolio policies, see our Editorial Policies and the Editorial Policy Checklist.

Statistics

For all statistical analyses, confirm that the following items are present in the figure legend, table legend, main text, or Methods section.

n/a	Confirmed	
☒	☐	The exact sample size (n) for each experimental group/condition, given as a discrete number and unit of measurement
☒	☐	A statement on whether measurements were taken from distinct samples or whether the same sample was measured repeatedly
☐	☒	The statistical test(s) used AND whether they are one- or two-sided<br>*Only common tests should be described solely by name; describe more complex techniques in the Methods section.*
☒	☐	A description of all covariates tested
☐	☒	A description of any assumptions or corrections, such as tests of normality and adjustment for multiple comparisons
☒	☐	A full description of the statistical parameters including central tendency (e.g. means) or other basic estimates (e.g. regression coefficient) AND variation (e.g. standard deviation) or associated estimates of uncertainty (e.g. confidence intervals)
☒	☐	For null hypothesis testing, the test statistic (e.g. F, t, r) with confidence intervals, effect sizes, degrees of freedom and P value noted<br>*Give P values as exact values whenever suitable.*
☒	☐	For Bayesian analysis, information on the choice of priors and Markov chain Monte Carlo settings
☒	☐	For hierarchical and complex designs, identification of the appropriate level for tests and full reporting of outcomes
☐	☒	Estimates of effect sizes (e.g. Cohen's d, Pearson's r), indicating how they were calculated

Our web collection on statistics for biologists contains articles on many of the points above.

Software and code

Policy information about availability of computer code

Data collection	No custom code was developed since the extraction of travel time was done through the Google Maps internal Directions API (https://developers.google.com/maps/documentation/directions/overview).
Data analysis	Not applicable

For manuscripts utilizing custom algorithms or software that are central to the research but not yet described in published literature, software must be made available to editors and reviewers. We strongly encourage code deposition in a community repository (e.g. GitHub). See the Nature Portfolio guidelines for submitting code & software for further information.

Data

Policy information about availability of data

All manuscripts must include a data availability statement. This statement should provide the following information, where applicable:

- Accession codes, unique identifiers, or web links for publicly available datasets
- A description of any restrictions on data availability
- For clinical datasets or third party data, please ensure that the statement adheres to our policy

Provide your data availability statement here.

Research involving human participants, their data, or biological material

Policy information about studies with human participants or human data. See also policy information about sex, gender (identity/presentation), and sexual orientation and race, ethnicity and racism.

Reporting on sex and gender	Not applicable
Reporting on race, ethnicity, or other socially relevant groupings	We utilised Meta's Relative Wealth Index (RWI) as a measure of wealth at the level of S2 cell39. This index estimates the relative wealth of the people living in each micro-region relative to others in the same country. The index is based on de-identified connectivity data, geospatial "big" data from satellites and other existing sensors, used to train a machine-learning algorithm that predicts microregional poverty. Data for Nigeria was based on a survey of 40,680 households in 899 unique survey locations (known as 'villages'). The survey included questions that assessed economic circumstances of each household. RWI values are available for small areas (also referred to as micro-regions), each of approximately 2.4km2 in size. RWI for each S2 cell was taken from that of its nearest micro-region.
Population characteristics	Not applicable
Recruitment	Not applicable
Ethics oversight	Ethics approval for the study was obtained from the National Health Research and Ethics Committee in Nigeria (NHREC/01/01/2007-11/04/2022) and the University of Greenwich Research and Ethics Committee (UREC/21.4.7.8).

Note that full information on the approval of the study protocol must also be provided in the manuscript.

Field-specific reporting

Please select the one below that is the best fit for your research. If you are not sure, read the appropriate sections before making your selection.

☐ Life sciences ☒ Behavioural & social sciences ☐ Ecological, evolutionary & environmental sciences

For a reference copy of the document with all sections, see nature.com/documents/nr-reporting-summary-flat.pdf

Behavioural & social sciences study design

All studies must disclose on these points even when the disclosure is negative.

Study description	Cross-sectional study
Research sample	Women of childbearing age (WoCBA) aged 15–49 years from WorldPop's open spatial demographic data portal.
Sampling strategy	Total population
Data collection	The study involved the assembly of data to define extended city boundaries to include peripheral suburbs, verify comprehensive emergency obstetric Care (CEmOC) facility functionality and geographic location (latitude and longitude), and map population distribution for women of childbearing age (WoCBA) aged 15–49 years to estimate the travel time to care.
Timing	Data collection for health facility verification took place from March to August 2022.
Data exclusions	No data were excluded from the analysis.
Non-participation	Not relevant
Randomization	Not relevant

Reporting for specific materials, systems and methods

We require information from authors about some types of materials, experimental systems and methods used in many studies. Here, indicate whether each material, system or method listed is relevant to your study. If you are not sure if a list item applies to your research, read the appropriate section before selecting a response.

Materials & experimental systems

n/a	Involved in the study
☒	☐ Antibodies
☒	☐ Eukaryotic cell lines
☒	☐ Palaeontology and archaeology
☒	☐ Animals and other organisms
☒	☐ Clinical data
☒	☐ Dual use research of concern
☒	☐ Plants

Methods

n/a	Involved in the study
☒	☐ ChIP-seq
☒	☐ Flow cytometry
☒	☐ MRI-based neuroimaging

Plants

Seed stocks	*Report on the source of all seed stocks or other plant material used. If applicable, state the seed stock centre and catalogue number. If plant specimens were collected from the field, describe the collection location, date and sampling procedures.*
Novel plant genotypes	*Describe the methods by which all novel plant genotypes were produced. This includes those generated by transgenic approaches, gene editing, chemical/radiation-based mutagenesis and hybridization. For transgenic lines, describe the transformation method, the number of independent lines analyzed and the generation upon which experiments were performed. For gene-edited lines, describe the editor used, the endogenous sequence targeted for editing, the targeting guide RNA sequence (if applicable) and how the editor was applied.*
Authentication	*Describe any authentication procedures for each seed stock used or novel genotype generated. Describe any experiments used to assess the effect of a mutation and, where applicable, how potential secondary effects (e.g. second site T-DNA insertions, mosiacism, off-target gene editing) were examined.*
